# High fidelity fibre-based physiological sensing deep in tissue

**DOI:** 10.1038/s41598-019-44077-7

**Published:** 2019-05-22

**Authors:** Tushar R. Choudhary, Michael G. Tanner, Alicia Megia-Fernandez, Kerrianne Harrington, Harry A. Wood, Adam Marshall, Patricia Zhu, Sunay V. Chankeshwara, Debaditya Choudhury, Graham Monro, Muhammed Ucuncu, Fei Yu, Rory R. Duncan, Robert R. Thomson, Kevin Dhaliwal, Mark Bradley

**Affiliations:** 1 Institute of Biological Chemistry, Biophysics and Bioengineering, School of Engineering & Physical Sciences, Heriot-Watt University, Edinburgh, UK; 2 EPSRC Proteus IRC Hub, Centre for Inflammation Research, Queen’s Medical Research Institute, University of Edinburgh, Edinburgh, UK; 3 SUPA, Institute of Photonics and Quantum Sciences, School of Engineering & Physical Sciences, Heriot-Watt University, Edinburgh, UK; 4 EaStChem, School of Chemistry, University of Edinburgh, Edinburgh, UK; 5 Centre for Photonics and Photonic Materials, Department of Physics, University of Bath, Bath, UK; 6 Present Address: The Roslin Institute and Royal (Dick) School of Veterinary Studies, University of Edinburgh, Edinburgh, UK

**Keywords:** Fluorescent dyes, Diagnostic markers, Imaging and sensing

## Abstract

Physiological sensing deep in tissue remains a clinical challenge. Here a flexible miniaturised sensing optrode providing a platform to perform minimally invasive *in vivo in situ* measurements is reported. Silica microspheres covalently coupled with a high density of ratiometrically configured fluorophores were deposited into etched pits on the distal end of a 150 µm diameter multicore optical fibre. With this platform, photonic measurements of pH and oxygen concentration with high precision in the distal alveolar space of the lung are reported. We demonstrated the phenomenon that high-density deposition of carboxyfluorescein covalently coupled to silica microspheres shows an inverse shift in fluorescence in response to varying pH. This platform delivered fast and accurate measurements (±0.02 pH units and ±0.6 mg/L of oxygen), near instantaneous response time and a flexible architecture for addition of multiple sensors.

## Introduction

Alterations in the physiological environment in tissues can drastically impact biological processes. Despite the presumption of tightly regulated levels of key physiological parameters such as [H^+^] and oxygen, in many areas of the human body the environmental physiology is unknown due to the paucity of miniaturised clinically compatible technologies. Thus, the aim of this study was to develop a flexible microendoscopic optrode for the accurate, robust and multiplexed sensing of pH and oxygen, which could be passed into remote regions of the human body. We demonstrate in this study that the distal alveolar acinar gas exchanging units of the lung can be accessed, where pH and oxygen play a critical role in maintaining homeostasis and are potential biomarkers of pathological processes, although clearly the platform technology is widely applicable to other regions.

**Figure 1:**
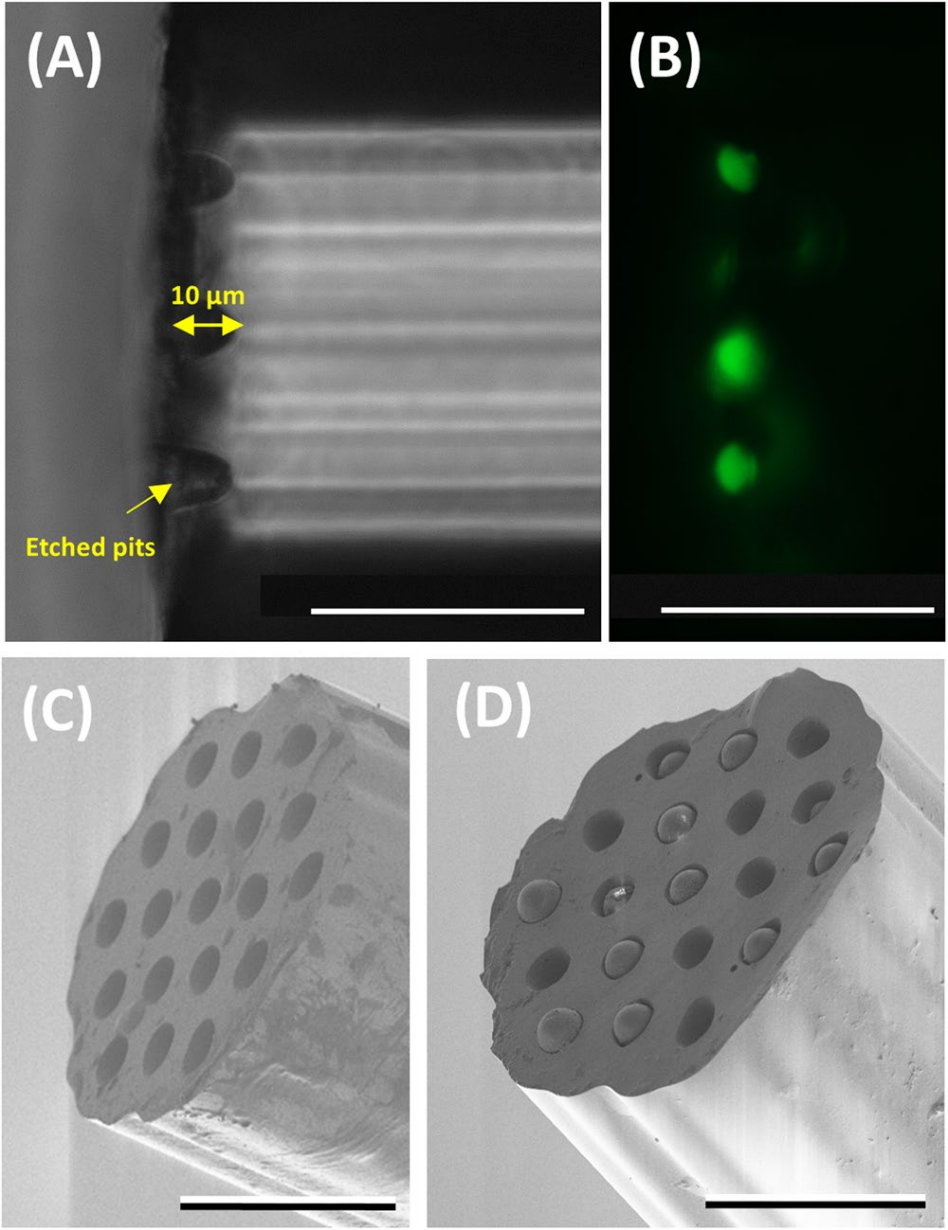
Optrode fabrication and characterisation. (**A**) Bright-field image of a fibre (viewed from the side) with etched pits (~10 µm in depth). (**B**) Fluorescence image (excitation 488 nm and emission 520 nm) of the etched fibres (viewed from the side) after the pH sensors (fluorescein-based) were loaded into the pits. Note the focal plane of image in (**B**) is different from (**A**) to highlight the loaded cores. (**C**,**D**) SEM images of an etched optical fibre before and after the addition of the microspheres. The scale bar in all the images is 50 µm.

The platform consists of novel pH sensors (fluorescein based) and oxygen sensors (palladium porphyrin complex based)^1^ covalently attached to silica microspheres (10 µm diameter) loaded into pits etched into the distal facet of a 19 core (10 µm core diameter) multicore fibre (total diameter of ~150 µm, see Fig. 1). Fluorescence is excited through selective coupling of light to a single core at the proximal end of the fibre, from which the spectrum is also measured, enabling multiplexing of sensors across the multi-core fibre.

## Results

### Deposition of pH and oxygen sensors on multicore sensing platform

Following the pioneering work of Walt^2^, fluorescent reporters covalently coupled to 10 µm amino modified silica microspheres were used as an optically robust solution to enable *in vivo* multiplexed pH and oxygen sensing. Thus, a bespoke multicore optical fibre (150 µm in diameter) with 19 germanium-doped cores matched to the diameter of the silica microspheres (10 µm) was fabricated and selectively etched with hydrofluoric acid, with the dopants directing the generation of concave pits aligned to the cores (see Fig. 1). Into these pits the 10 µm silica microspheres were firmly and irreversibly deposited. This enabled each core to act as an isolated independent measurement channel, enabling multiparametric sensing through the specific illumination of different cores.

An important property of the amino modified silica microspheres is the local concentration of 1 × 10^9^ free amino groups to which fluorescent sensors can bind. If all the sites are loaded with fluorophores, then the spacing between adjacent fluorophores would be ~5 Å. At this loading density following illumination, fluorophores would become excited with subsequent emission resulting in energy transfer with proximal neighbours (e.g. FRET) and as shown below this phenomenon which has never been previously reported, dramatically improves the performance of sensors.

**Figure 2:**
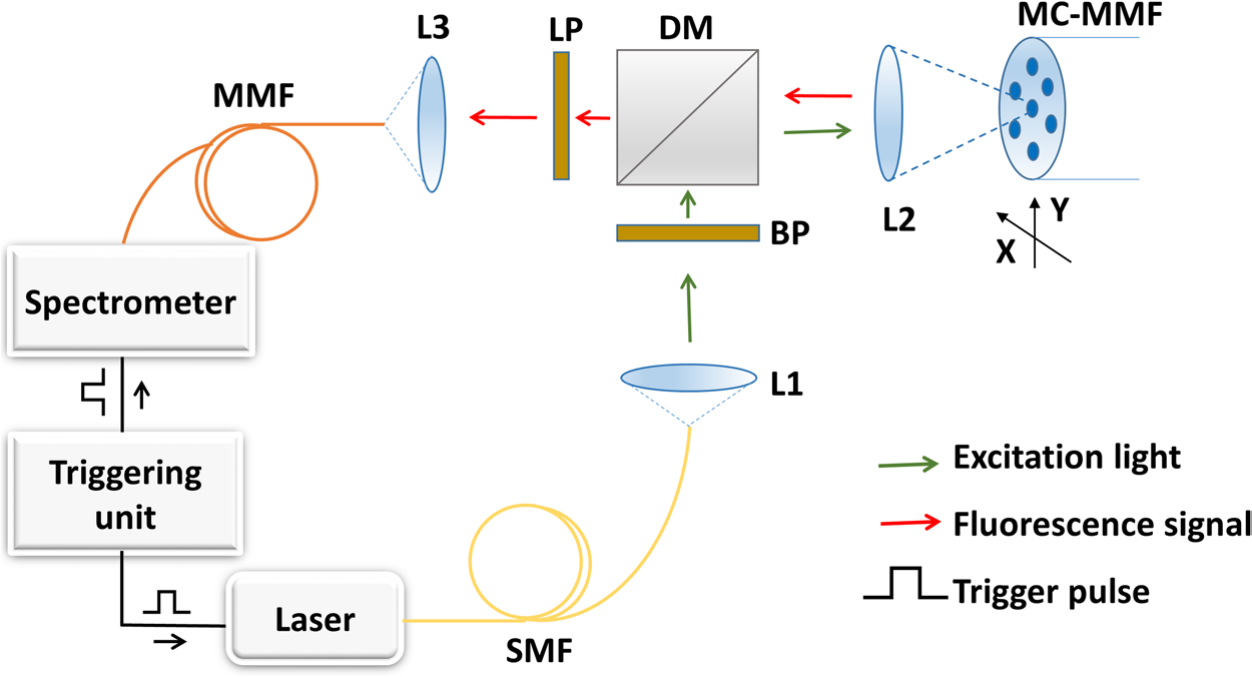
Optical setup to allow fibre based sensing. A 520 nm laser source (10 µW power and 100 ms exposure) was used as a pump source to excite both the pH and oxygen sensors. The pump illumination was launched from a single mode fibre (SMF) collimated by an aspheric lens (L1), passing through a band pass filter (BP) removing unwanted long wavelength light, and reflected by the dichroic mirror (DM), then focused with an identical aspheric lens (L2) and selectively coupled into the fibre core (with XY control of the sensing fibre mount). The returned light from the fibre core was collimated by L2, passed through the dichroic mirror and a long pass filter (LP, removing any reflected pump light). The fluorescence was then focused by lens L3 into a 50 µm core multimode fibre (MMF) patch cable, and directed to a spectrometer (Ocean Optics QEPro). The triggering unit (TTL pulse generator) controlled both laser and spectrometer allowing on demand short integration time (100 ms) synchronised measurements.

Optrode measurements were performed using an epi-fluorescence arrangement (see Fig. 2) with light selectively coupled into a single chosen core.

#### Silica microspheres loaded with fluorescent reporters for oxygen and pH sensing

**Figure 3:**
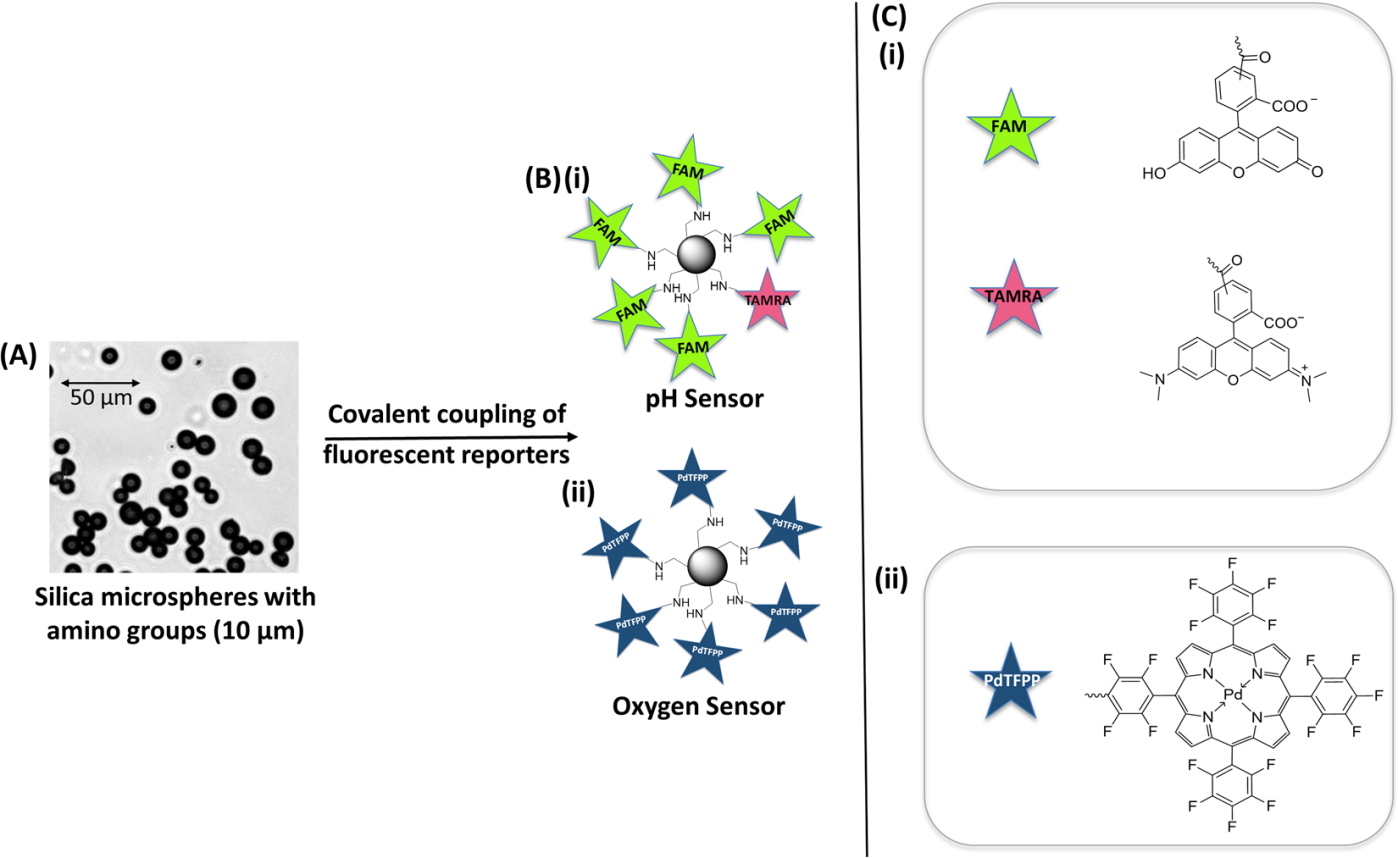
Sensor fabrication. (**A**) Brightfield image of 10 µm amino functionalised silica microspheres. (**B**) (i) pH sensor: 5(6)-Carboxyfluorescein (FAM) and 5(6)-Carboxytetramethylrhodamine (TAMRA) covalently coupled onto the silica microspheres (not representative of loading ratio). (ii) Oxygen sensor: palladium(II) 5,10,15,20-meso-tetrakis-(2,3,4,5,6-pentafluorophenyl)-porphyrin (PdTFPP) covalently coupled onto the silica microspheres. (**C**) The structures of: (i) FAM and TAMRA and (ii) PdTFPP for conjugation onto the silica microspheres.

A widely used luminescent reporter of dissolved oxygen is the Palladium(II) 5,10,15,20-meso-tetrakis-(2,3,4,5,6-pentafluorophenyl)-porphyrin) complex (PdTFPP) (Fig. 3), that has good quantum yield and displays robust photostability^3,4^. PdTFPP absorbs between 400 nm and 560 nm, with fluorescent emission between 660–800 nm^5^. PdTFPP displays high fluorescence emission intensity in the region λ > 640 nm at low oxygen levels and the fluorescence levels reduce as oxygen levels increase. Importantly the fluorescent spectrum of PdTFPP has a non-variant region between 550 nm to 640 nm enabling ratiometric measurements (see Ratiometric sensing section). An excitation wavelength of 520 nm was used, which was suitable for excitation of the pH sensors discussed below and also yielded reduced fibre auto fluorescent background signals.

**Figure 4:**
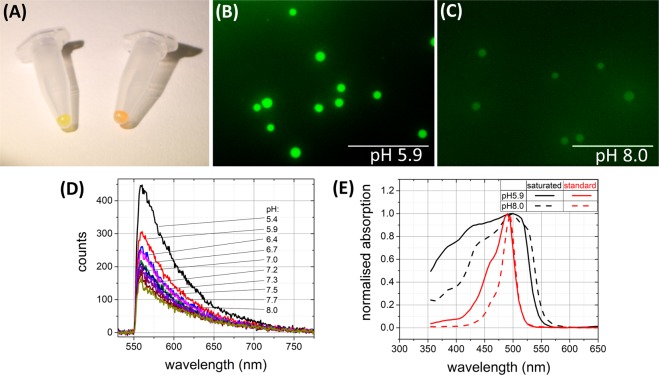
Comparison of different pH sensors. (**A**) Image of “standard” loaded (left) and “saturated” loaded (right) pH sensors (note the colour difference). (**B**,**C**) “Saturated” pH sensors imaged via a fluorescence microscope at pH 5.9 (**B**) and pH 8.0 (**C**) (scale bar = 100 µm). (**D**) Emission spectra from the “saturated” pH sensors at different pH. (**E**) Absorption spectra of the “saturated” and normally loaded pH sensors at pH 5.9 and 8.0.

Fluorescein is known to show increased emission intensity with increasing pH^6,7^ which has led to fluorescein being a widely applied pH sensor in a variety of platforms^8–12^. The amino modified silica microspheres were saturation coupled with 5-(6) carboxyfluorescein (FAM) (see Methods). However, contrary to all literature reports on fluorescein pH response, the microsphere sensors exhibited a totally unexpected decrease in emission with increasing pH (Fig. 4), confirmed with conventional fluorescence microscopy of microspheres in pH buffers (Fig. 4B,C). Interestingly, when the loading was reduced (see the SI section for comparison) the sensors behaved as per literature expectations (SI Fig. S1).

Sensors with different loading densities were observed to exhibit significantly different absorption spectra, with the microspheres displaying (macroscopically) very different properties (Fig. 4A). The “saturated” microspheres exhibited absorption features at shorter wavelength (Fig. 4E) suggesting, perhaps, the presence of dimer and/or trimers of fluorescein^13^. The “saturated” pH sensors showed lower emission intensity than the “standard” loaded sensors (see SI, Fig. S1) due to an enhanced internal quenching effect since the dye molecules are in such close proximity on the microspheres. In addition, corresponding longer wavelength emission was observed for the “saturated” loaded pH sensors (see Fig. S1). The “saturated” loaded pH sensors were also observed to be more robust to photo-bleaching (see SI, Fig. S2), perhaps due to the promoted relaxation mechanisms between molecules in close proximity.

The fibre optrode utilising the “saturated” loaded FAM based pH sensor was observed to show a strong response to changes in pH and be much more robust than the “standard” loaded sensor. However, the intensity of a single fluorescent signal alone is always likely to be an unreliable measurement in a complex imaging or biological system, and thus a novel self-referencing pH sensor (details in next section) was designed.

### Ratiometric sensing

**Figure 5:**
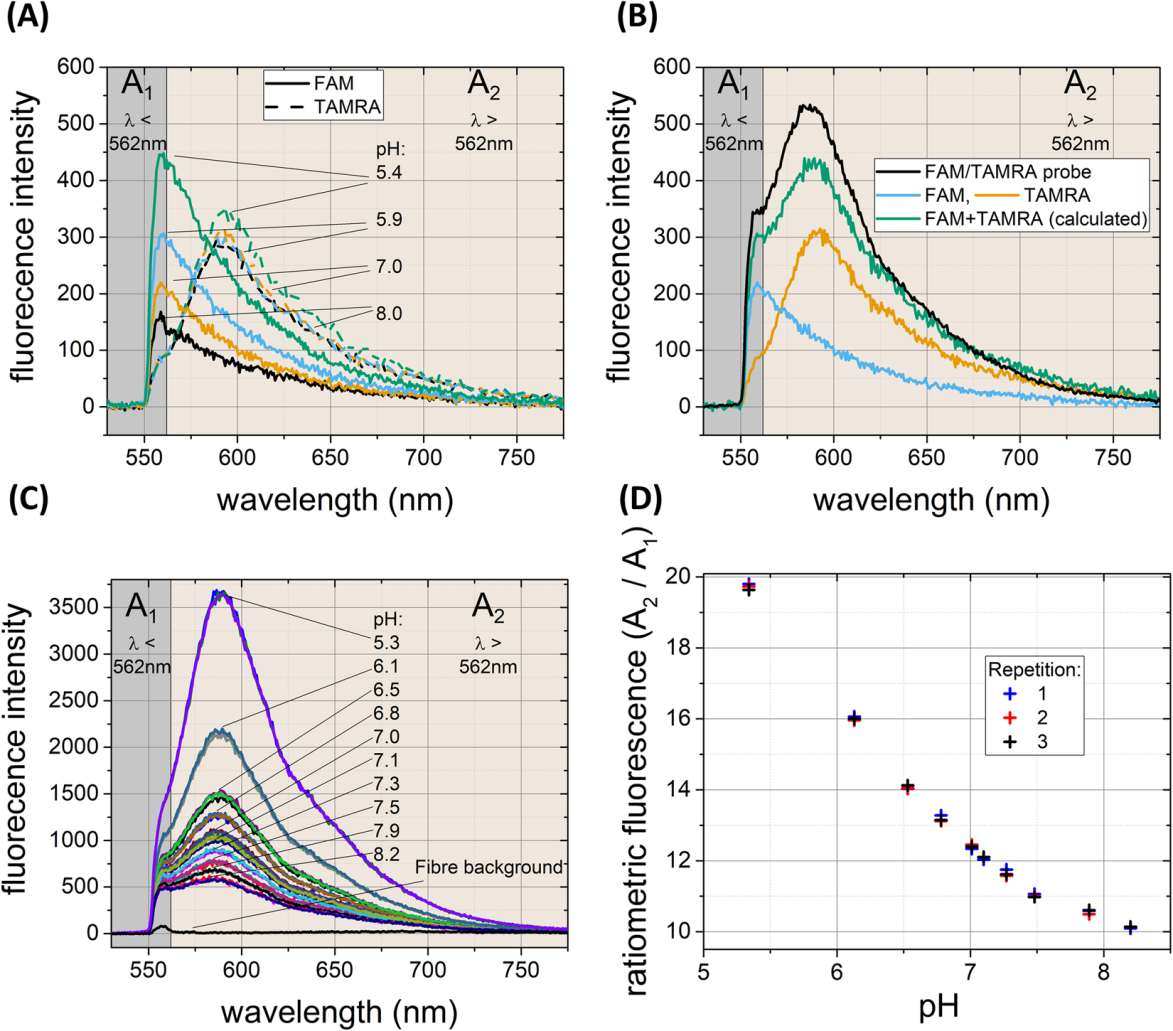
pH response of the optrode and separate fluorophores. (**A**) Fluorescence emission spectra of “saturated” loaded FAM (solid lines) and TAMRA (dotted lines) microspheres at different pH. (**B**) Fluorescence emission spectra of FAM only (blue line) and TAMRA microspheres (orange line) at pH 7. Fluorescence emission spectra at pH 7 of FAM and TAMRA microspheres when numerically combined (green line) is compared to the spectral shape of the fabricated FAM/TAMRA probe (black line, halved in amplitude to aid comparison). (**C**) Fluorescence emission spectra of the FAM/TAMRA pH sensor at different pH. The FAM dominant region is highlighted as A1 (λ < 562 nm) and TAMRA dominant region as A2 (λ > 562 nm). (**D**) Calculated ratio of fluorescence intensity from the measured spectra at different pH (n = 3). The ratio was calculated by dividing the area under the curve of spectral region A2 by A1. All measurements were from a single sensor from one core of the optrode at 520 nm (Power 10 µW and 100 ms exposure time). Uncertainty in measurement was calculated to be ±0.02 pH units (standard deviation, see methods).

5(6)-Carboxytetramethylrhodamine (TAMRA) was attached along with 5(6)-Carboxyfluorescein (FAM) (Fig. 3), with the ratio optimised to allow observation of both spectra (see Fig. 5). The spectral ratio to determine pH was calculated by dividing the number of counts in the TAMRA dominant region of the spectra (A2) by the number of counts in the FAM dominant region of the spectra (A1) (Fig. 5). This fluorophore combination is a known FRET pair^14^ and thus in combination with the “saturated” loading of both fluorophores leads to a complex relationship in the observed spectra, with the TAMRA fluorescent acceptor intensity changing in response to the fluorescein levels. The resulting dependence on pH of the combined spectral features differs in spectral regions dominated by the TAMRA (A2 region) or FAM (A1 region) in Fig. 5. Figure 5A shows the fluorescence intensity response of the “saturated” loaded FAM only pH sensor and TAMRA microsphere at different pH. Only the FAM spectrum shows a significant change in intensity in response to pH.

In Fig. 5A,B it is apparent that the FAM spectra dominates region A1, while TAMRA dominates in region A2. When the fluorescence spectra of FAM and TAMRA were numerically combined (Fig. 5B, green line), the shape of the spectrum matched the FAM/TAMRA loaded probe (black line). The spectral signatures of the fluorophores were not fully resolvable from each other; however, a dominant peak was observed in the A2 region, originating from the TAMRA, with a “shoulder” in the A1 region from the FAM.

#### pH measurements

The FAM/TAMRA optrode response to pH is shown in Fig. 5C,D, with the analysed spectral ratio demonstrating responses that are robust, reversible and reliable to changes in pH with minimal variation between repetitions.

#### Oxygen concentration analysis

**Figure 6:**
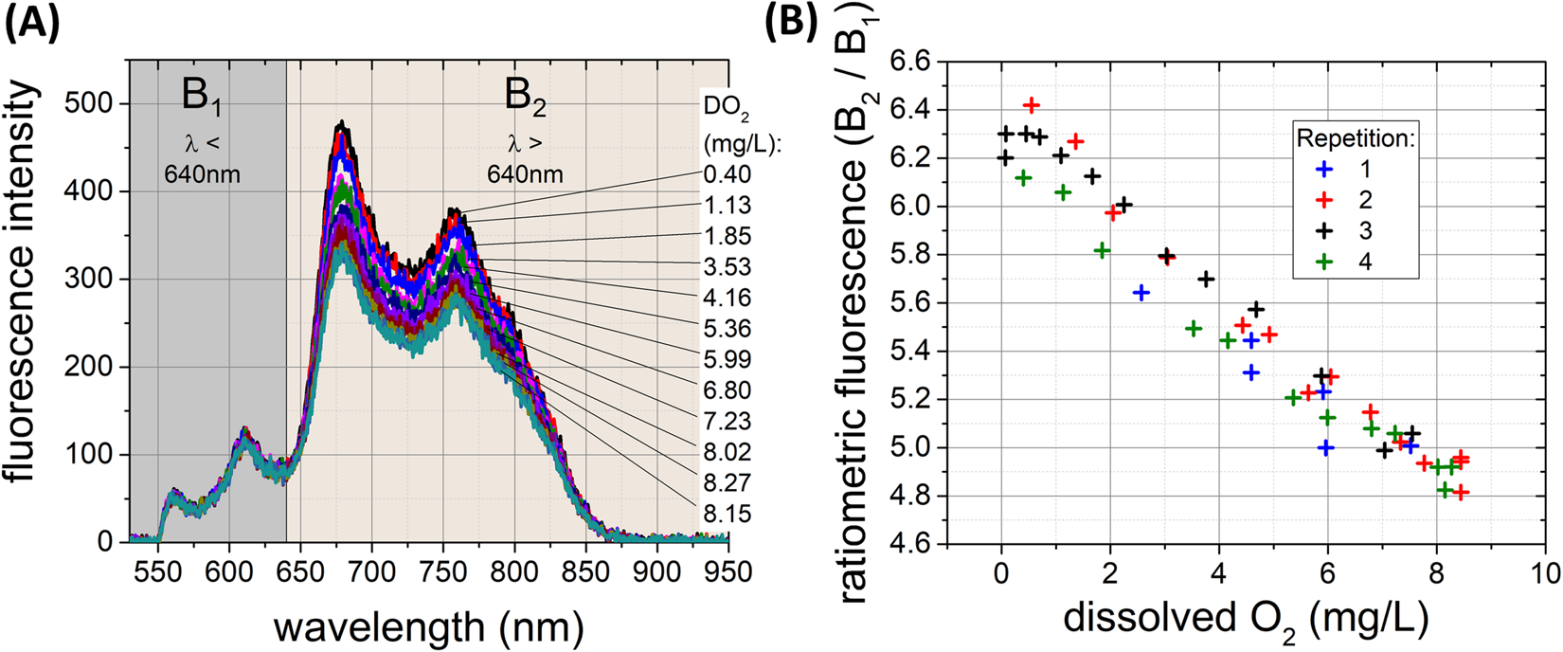
Response of the optrode to varying concentrations of dissolved oxygen. The optrode response was measured in distilled water changing the dissolved oxygen level from 8 mg/L (air saturated) to near full depletion (~0 mg/L). (**A**) Fluorescence emission spectra at different dissolved oxygen. The non-variant region is highlighted as B1 (λ < 640 nm) and variant region as B2 (λ > 640 nm). (**B**) Calculated ratio of fluorescence intensity from the measured spectra at different oxygen concentrations (n = 4). The ratio was calculated by dividing area under the curve in spectral region B2 by B1. All measurements were from a single sensor from one core of the optrode at 520 nm (Power 10 µW and 100 ms exposure time). Uncertainty in measurement was calculated to be ±0.6 mg/L (standard deviation, see methods).

The oxygen optrode (Palladium porphyrin complex) was used to measure dissolved oxygen concentrations with Fig. 6A showing the emission spectra of the oxygen sensor at various dissolved oxygen levels.

As previously noted, one region of the spectra was stable in fluorescence intensity with different oxygen concentrations, whilst another region changed with varying oxygen concentrations (highlighted as B1 and B2 in Fig. 6A). The ratiometric response (B2/B1) is shown in Fig. 6B.

### Application in a perfused and ventilated *ex vivo* ovine lung model

**Figure 7:**
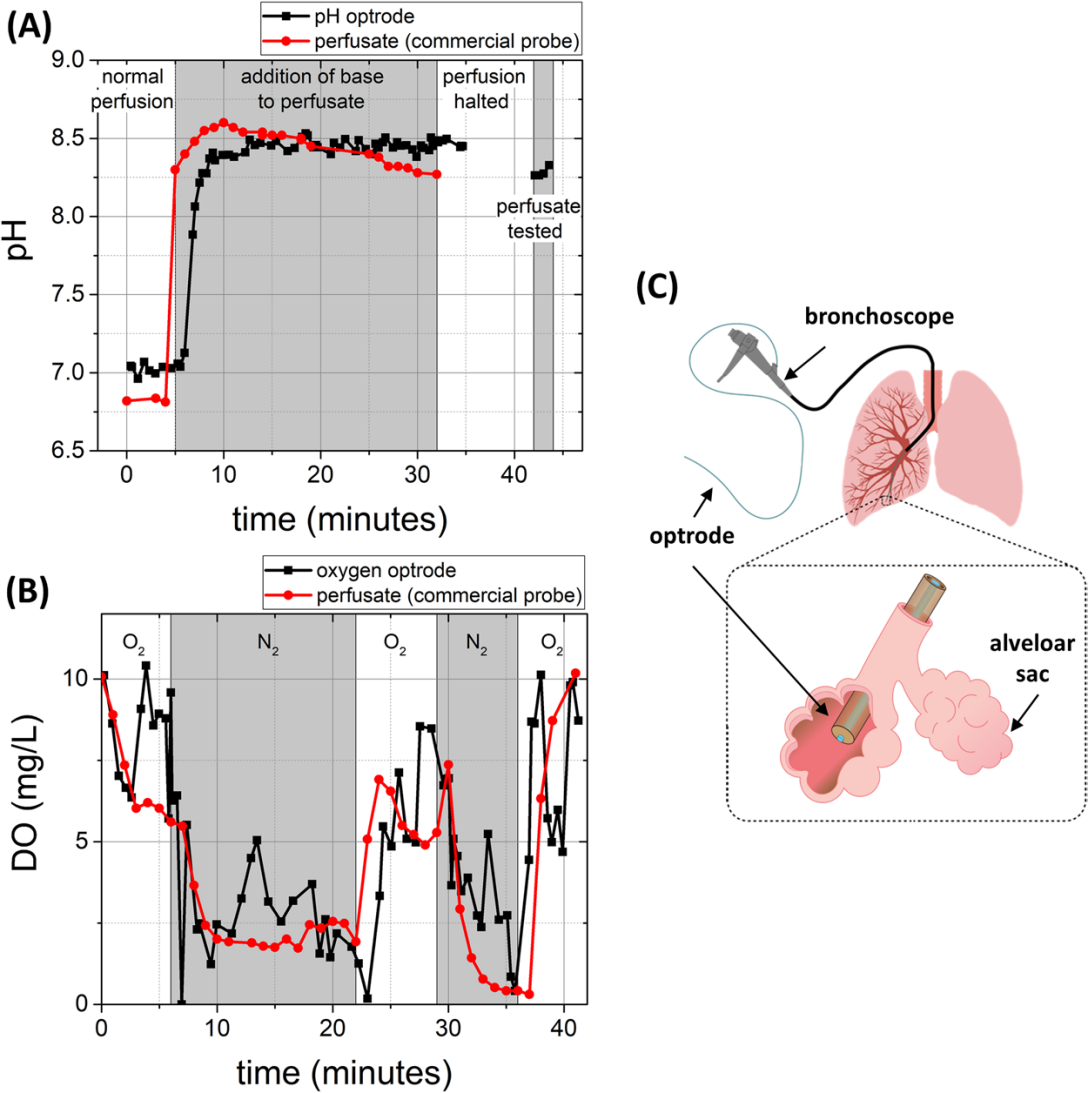
Optrode measurements of pH and O_2_ levels in ventilated and perfused ovine lungs from two animals. (**A**) The comparison of pH measurements taken with the optrode (black line) in the alveolar space and of the perfusate recorded with a conventional pH meter (red line) with pH modulation of the perfusate (addition of base). The data points on the right are from the optrode, which has been removed from the lung and placed in the perfusate. (**B**) Comparison of the dissolved oxygen measurement as determined with the optrode (black line) in the alveolar space and of the perfusate recorded with a commercial meter (red line), with switching of ventilated gas mixtures. (**C**) Placement of the optrode in the alveolar sac of the distal lung.

The optrode was applied in a ventilated and perfused *ex vivo* ovine lung model^15^, with the fibre optrode passed trans-bronchially into the distal alveolar space as shown in Fig. 7. The perfusate was monitored for pH or O_2_ using commercial meters while simultaneous measurements of the analyte were acquired with the optrode in the alveolar space throughout the duration of the experiment, with the pH or oxygen level of the circulating perfusate readily tuneable by the addition of base or altering the O_2_/N_2_ ratio in the ventilation circuit (see Fig. 7). While the optrode measured local changes in the distal lung, the correlating perfusate measurements were external to the organ and thus experience a differing but related response (see Methods section). The optrode pH measurements correlated well with the commercial pH meter demonstrating its robustness and sensitivity in the whole lung model (see Fig. 7A). As expected, some difference was observed between alveolar tissue pH and perfusate pH, including a delay in optrode pH response following changes in perfusate pH due to the circulation time and local tissue buffering. The optrode O_2_ measurements and the commercial meter also showed a good correlation to changes in the ventilated gas mixture (see Fig. 7B). As the lungs were undergoing ventilation with normal room air in addition to external gases, it is possible the fluctuations are representative of the true environment changing with each breath. Both optrode and perfusate measurements experience some delay in response to ventilation changes due to gas transfer and circulation time.

### Optrode robustness

**Figure 8:**
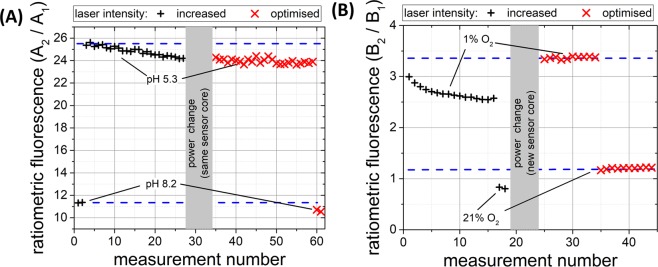
Measurement stability with repeated illumination and varying power. (**A**) Repeated measurement of pH with 20 µW illumination (black crosses), then reduced to 10 µW (red crosses). (**B**) Repeated measurement of oxygen concentration at 100 µW illumination (black crosses), then reduced to 10 µW (new sensor core, red crosses). Blue dashed lines have been added to act as a guide to the data for the unbleached sensor responses.

To demonstrate the photostability and reproducibility of the pH sensor, repeated measurements were taken using pH buffers (Fig. 8A). The laser power was increased to allow some degradation of ratiometric fluorescence (20 µW, double the optimal power, black crosses Fig. 8A), then reduced to 10 µW (optimised measurement power, red crosses Fig. 8A). While at increased power (black crosses) some sensor degradation was observed, at normal operating conditions (red crosses) photobleaching was minimal over the space of 25 measurements (Fig. 8A). See SI for investigation of the robustness of FAM “standard” and “saturated” loaded microspheres.

The ratiometric nature of the sensor means that it is naturally resistant to fluctuations in input laser power. Notably as the same sensor core was used throughout the above investigation we also demonstrate this principle in which the ratiometric fluorescence value was unaffected by the 2-fold reduction in laser pump power, much greater than the fluctuations in the laser or optical coupling expected in normal operation.

Similar tests were performed on the oxygen sensor to demonstrate photostability. To observe degradation, in this case, a greater increase in the input laser power was necessary to degrade this stable ratiometric fluorescence sensor. When a 10-fold higher illumination power was applied (100 µW), some degradation of ratiometric fluorescence was observed over 16 measurements (black crosses) shown in Fig. 8B. Reducing power to optimal levels (10 µW) confirmed no observable photobleaching effect on the measurement result from a fresh sensor core (red crosses) over 20 repetitions (10 at depleted and 10 at room air oxygen levels).

## Discussion

This work demonstrates high fidelity measurement of pH and oxygen using a miniaturised flexible fibre based optrode. Exceptional measurement sensitivity was achieved for a miniaturised optrode, with an accuracy of the pH sensor of 0.02 pH units and oxygen sensor accuracy of 0.6 mg/L (for calculation see Methods: Data processing and measurement uncertainty). Response to pH and oxygen environments were both observed to be instantaneous (less than 1 second) when the optrode was moved between liquids. Ratiometric pH measurements ensured robustness and resistance to the errors typical of intensity-only based measurements e.g. photobleaching and pump power fluctuations. For the first time, we demonstrated that carboxyfluorescein covalently coupled to silica microspheres at high density exhibits an anomalous dependency of fluorescence intensity with pH, with increased pH reducing the fluorescence yield. Utilising triggered low power illumination and synchronised measurement, the fluorescent sensors are observed to be robust to photobleaching. The single miniaturised multicore fibre multiplexing approach demonstrated here offers the possibility of both pH and oxygen measurements *in vivo* at previously inaccessible sites. Thus, we demonstrated the use of the optrode in a ventilated and perfused *ex vivo* ovine lung model, deployed into the air sacs, with both pH and oxygen sensors responding well to the change of pH and oxygen in the lung.

There are many reported optical fibre based sensors for either pH^8,9,16–20^ or oxygen^21,22^ which utilise trapping of nanoparticles/microspheres or sensor dye molecules on the fibre facet using suitable coatings such as sol-gel or polymers. The use of optical fibre in these techniques provides flexibility, but the sensors often have slow response times (in minutes) or limited accuracy and range. The use of a fluorescent polymer coating over an entire fibre facet is incompatible with precise deposition. Recently, two techniques for dual sensing of pH and oxygen have been described by the same group^23,24^, in which a mixture of pH and oxygen sensing microspheres were deposited on the fibre using suitable sol-gel or hydrogel coating. The results demonstrated dual sensing, although the response time reported was slow (1.5–2 minutes for pH and ~30 seconds for oxygen) due to the coating deposition method. The multiplexed sensing platform described in this study overcomes these issues, and provides fast, stable and accurate measurements, near instantaneous response time and a flexible architecture for addition of multiple sensors.

The use of multicore fibre also provides a highly flexible architecture. The work described here uses only two different sensors, but the number of sensors can be increased easily with availability and requirement. The multiplexed fibre sensor assembly process is simple, low effort, robust, reliable, and the loaded microspheres self locate. The size of microspheres, size or number of fibre cores, or the size of the fibre itself can be very easily modified or adapted depending on the application requirement. The chemical loading of sensors (fluorophores) onto silica microspheres can simply be performed in a controlled, efficient and repeatable environment before being introduced to the optical fibres.

After successful demonstration of the pH and oxygen optrode in *ex vivo* lung models, the next step will be to validate this technology through clinical translation, which will require packaging of the optrode in suitable biocompatible materials. Crucially, the optrode has been demonstrated to survive standard sterilisation procedures (see SI, Fig. S3).

Our current understanding of key physiological parameters (pH, oxygen etc.) in health or in disease comes from invasive studies on animals^25^ and cannot be applied to patients in clinic, either due to the invasive nature of the measurement or the lack of suitable techniques. The work described here is a significant step forward in the direction of clinical application of *in vivo* sensing in previously inaccessible areas of the human body which will help build our understanding of physiological parameters in disease pathology.

## Methods

### Preparation of pH sensors

#### FAM/TAMRA pH sensor

Amino functionalized silica microspheres (10 μm, Kisker Biotech Gmbh & Co., 25 mg, 0.5 mL) were centrifuged (5 min, 10,000 RPM), washed with MeOH and centrifuged (X3, 5 min, 10,000 RPM), washed with ether and centrifuged (x1, 5 min, 10,000 RPM) and left to dry at 40 °C. A solution containing 5(6)-Carboxyfluorescein (FAM)/5(6)-Carboxytetramethylrhodamine (TAMRA) in a molar ratio of 300:1 (11.3 mg of 5(6)-Carboxyfluorescein and 0.04 mg of 5(6)-Carboxytetramethylrhodamine) and Oxyma (4.2 mg) in dimethylformamide (DMF, 0.42 mL) were stirred (200 RPM) for 10 min, then N,N′- diisopropylcarbodiimide (DIC, 4.5 μL, 0.03 mmol) was added and after 1 minute the solution was added to the microspheres. The reaction was kept in a thermal mixer (2 hours, 50 °C, 300 RPM). The reaction mixture was centrifuged (5 min, 10,000 RPM) and the supernatant carefully removed. The microspheres were washed with DMF and the coupling reaction was repeated three times. Finally, the microspheres were washed sequentially with DMF, 20% Piperidine/DMF, DMF, MeOH, ether and dried at room temperature.

#### “Saturated” and “standard” loaded pH sensor

The preparation method for the “saturated” loaded FAM only pH sensor was the same as above except that only FAM (11.4 mg) was used. In the case of the “standard” loaded pH sensor all the solutions were diluted by 10×.

#### TAMRA loaded microspheres

The same method as above for the FAM/TAMRA pH sensor was followed, except only TAMRA (13 mg) was used.

### Preparation of oxygen sensors

Amino functionalized silica microspheres (10 μm, Kisker Biotech Gmbh & Co., 50 mg, 0.5 mL) were centrifuged (5 min, 10,000 RPM), washed with MeOH and centrifuged (X3, 5 min, 10,000 RPM), washed with ether and centrifuged (x1, 5 min, 10,000 RPM) and left to dry at 40 °C. 2 mg of PdTFPP (Sigma-Aldrich) and 50 mg of the microspheres were dispersed in 1 mL of diphenylether (Sigma-Aldrich). The reaction was heated under N_2_ at 230 °C in a vial for 3 hours. After cooling to room temperature, the mixture was centrifuged (5 min, 10,000 RPM) and the supernatant carefully removed. The microspheres were washed five times with dichloromethane and dried at room temperature.

### Multicore multimode fibre fabrication

The multicore multimode fibre was fabricated using the “stack and draw” process commonly used to fabricate photonic crystal fibres as described in detail elsewhere^26^. In brief, a germanium doped optical fibre preform (diameter = 32 mm, peak numerical aperture = 0.3, Draka-Prysmian) with a parabolic refractive index profile and a thin pure silica jacket was drawn down to rods (ø = 5.75 mm). To increase core to core separation in the final fibre, each of the rods was further jacketed with a pure silica tube (outer diameter = 10 mm), and drawn down to a diameter of 2.4 mm. 19 rods of this material were then stacked in a hexagonal close-packed array, placed into a jacket tube, and drawn down under a vacuum to form the final 19 core fibre and the coating was added to the fibre. The final diameter of the cores was 10 µm, with a centre-to-centre separation of 23 µm. The outer diameter of the fibre was 150 µm.

### Optrode fabrication

The multicore fibre (19 cores, core diameter 10 µm) after removing the coating with a razor blade was cleaved to give the fibre flat ends. One end of the fibre was etched using 40% hydrofluoric acid (HF) for 60 seconds. After etching, the end was sonicated in deionised water for 5 minutes to remove any HF from the cores. The etching parameters were optimised to produce pits with depths of ~10 µm to match the silica microspheres. The etched end of the fibre was then inserted into an eppendorf tube containing the silica microsphere loaded sensors (pH and oxygen) and gently tapped to load them onto the pits. The ends were then wiped using a tissue to remove any excess sensors. None of the microspheres were observed to have been lost during normal usage during the experiments described. Attempts to aggressively remove microspheres from the optrode by treatments of one minute of ultrasonic agitation in isopropanol showed approximately 90% retention.

### pH buffers

The pH buffers were prepared using a literature based protocol^15^ with the buffer pH checked using a commercial pH meter (SevenGo Duo Pro, Mettler Toledo, U.K.).

### Dissolved oxygen variation

Oxygen concentration was measured in water saturated with 20% oxygen (~8 mg/L) down to full depletion (0 mg/L). The concentration of oxygen in the distilled water was changed (between 0–8 mg/L) by bubbling either nitrogen or oxygen gas, and measured with a commercial O_2_ meter (SevenGo Duo Pro, Mettler Toledo, U.K.) for every measurement taken with the optrode.

### *Ex Vivo* ovine lung model

Ovine lungs for the experiments described were from ewes destined for cull and were euthanized under Schedule 1 of Animals (Scientific Procedures) Act 1986, in an approved facility at the University of Edinburgh, performed in accordance with relevant guidelines and regulations. After barbiturate overdose and transection of major neck vessels, the trachea was dissected and clamped *in situ* and the heart and lungs were removed “en bloc”. On the backtable, the heart was dissected off, leaving an open left atrium and a long section of pulmonary artery which was cannulated. The lungs were flushed with 2 litres of cold 0.9% saline with Heparin (2500 IU/L), retrograde flush via the pulmonary veins was then performed with a further 1 litre of cold saline. A 9 mm endotracheal tube (Rusch) was inserted into the trachea, secured and clamped with the lungs partially inflated. Lungs were then stored on ice for transportation and kept in a refrigerator (0–6 °C).

The *ex vivo* lung perfusion circuit consisted of an organ chamber to house the lungs connected to a hardshell reservoir (Maquet). The perfusate was circulated using a centrifugal pump (Maquet Rotaflow) back to the lungs via a gas membrane (Maquet) which was connected to a heat exchanger (Chalice Medical). The perfusate was delivered to the pulmonary circulation using a pulmonary artery catheter (XVIVO) with inline pressure sensing.

Once the lungs were transferred to the organ chamber 2 litres of room temperature perfusate (Phosphate Buffered Saline (PBSS, Gibco) supplemented with 10% Foetal bovine serum (FBS, Gibco) and 10000 IU Heparin) were circulated. The pulmonary artery catheter was secured in the pulmonary artery using a silk tie and a digital temperature probe was sutured into a pulmonary vein. The lungs received a retrograde flush on the circuit to de-air the pulmonary artery cannula which was then connected to the circuit.

The heater was switched on and gentle flow was commenced at 0.25 L/min and increased gradually over 20 minutes to the desired flow rate (at least 50% estimated cardiac output; 70 ml/kg/min) ensuring that the pulmonary artery pressure remained below 20 mmHg. Once the temperature of the effluent perfusate had reached 32 °C, protective volume controlled ventilation was commenced at a tidal volume of 300 ml, positive end expiratory pressure 5, rate 8 beats per minute.

Bronchoscopy was performed once ventilation had commenced and a biocompatible Pebax tube (length 1.5 m) (Vention Medicals, U.S.) with an inner diameter of 1 mm and outer diameter of 1.4 mm was passed by transbronchial puncture to the alveolar space to a dependent segment of lung allowing deployment of the optrode.

An alkali solution (pH 8.5) was prepared using 40% Trizma base (Sigma-Aldrich) in water for biasing of perfusate pH. N_2_ and O_2_ gases were introduced into the ventilation circuit (in addition to continued normal ventilation) to bias the respiratory oxygenation. In both cases the commercial probe was placed in the perfusate reservoir for comparison, but would be expected to show some variation from the very local changes measured/analysed by the optrode in the distal lung.

### pH measurements in lung model

The optrode was calibrated pre and post experiment in the pH buffers. The perfusate pH was tested directly with the optrode after removal from the lung, which showed good agreement with the commercial pH meter at the end of perfusion (Fig. 7).

### Oxygen measurements in lung model

The optrode was calibrated in water (20% oxygenated and depleted) pre and post experiment to correlate to a dissolved oxygen scale. A constant fluorescent background from the lung tissue contributing an offset in the calculated dissolved oxygen value was subtracted such that dissolved oxygen (compared to the minimum observed) is plotted.

### Data processing and measurement uncertainty

Fluorescent spectra are analysed with simple code (Matlab). In the case of the O_2_ spectra a fibre background was subtracted to better observe the signal and reference peak magnitude. Accuracy of measurement is calculated as follows. For pH measurement, as replicates were possible at repeatable pH values, standard deviation between the 3 replicates in Fig. 5D were calculated. In the region pH 6.5 to 7.5 the mean standard deviation was found. This was divided by the gradient of the response in this region (approximated as linear) to transpose to an error estimate on the x-axis, in pH units. For O_2_ measurement, as it was not possible to precisely recreate a particular dissolved oxygen level, a slightly different approach was used. The ratio response in Fig. 6 was approximated as linear, fitted, and the residuals found. The standard deviation of the residuals was calculated. This was transposed to the x-axis as for the pH measurement. As such both error calculations are quoted as the 1-sigma error.

## Supplementary information

Supplementary Dataset 1

## Data Availability

The experimental data is  available via Edinburgh DataShare at 10.7488/ds/2546.

## Code Availability

The code is available via Edinburgh DataShare at 10.7488/ds/2546.

## References

[1] Müller BJ, Burger T, Borisov SM, Klimant I (2015). High performance optical trace oxygen sensors based on NIR-emitting benzoporphyrins covalently coupled to silicone matrixes. Sensors and Actuators B: Chemical.

[2] Michael KL, Taylor LC, Schultz SL, Walt DR (1998). Randomly Ordered Addressable High-Density Optical Sensor Arrays. Analytical Chemistry.

[3] Borisov SM, Lehner P, Klimant I (2011). Novel optical trace oxygen sensors based on platinum(II) and palladium(II) complexes with 5,10,15,20-meso-tetrakis-(2,3,4,5,6-pentafluorphenyl)-porphyrin covalently immobilized on silica-gel particles. Analytica Chimica Acta.

[4] Koren K, Borisov SM, Klimant I (2012). Stable optical oxygen sensing materials based on click-coupling of fluorinated platinum(II) and palladium(II) porphyrins—A convenient way to eliminate dye migration and leaching. Sensors and Actuators. B, Chemical.

[5] Quaranta M, Borisov SM, Klimant I (2012). Indicators for optical oxygen sensors. Bioanalytical Reviews.

[6] Martin MM, Lindqvist L (1975). The pH dependence of fluorescein fluorescence. Journal of Luminescence.

[7] Mordon S, Devoisselle JM, Maunoury V (1994). *in vivo* pH measurement and imaging of tumor tissue using a pH-sensitive fluorescent probe (5,6–carboxyfluorescein): instrumental and experimental studies. Photochemistry and photobiology.

[8] Dafu C (1993). Optical-fibre pH sensor. Sensors and Actuators B: Chemical.

[9] Islam S, Bidin N, Riaz S, Krishnan G, Naseem S (2016). Sol–gel based fiber optic pH nanosensor: Structural and sensing properties. Sensors and Actuators A: Physical.

[10] Stich MIJ, Fischer LH, Wolfbeis OS (2010). Multiple fluorescent chemical sensing and imaging. Chemical Society Reviews.

[11] Bradley M (2008). pH sensing in living cells using fluorescent microspheres. Bioorganic & Medicinal Chemistry Letters.

[12] Han J, Burgess K (2010). Fluorescent Indicators for Intracellular pH. Chemical Reviews.

[13] Rohatgi KK, Singhal GS (1966). Nature of Bonding in Dye Aggregates. The Journal of Physical Chemistry.

[14] Caruso F, Donath E, Möhwald H (1998). Influence of Polyelectrolyte Multilayer Coatings on Förster Resonance Energy Transfer between 6-Carboxyfluorescein and Rhodamine B-Labeled Particles in Aqueous Solution. The Journal of Physical Chemistry B.

[15] Choudhury D (2017). Endoscopic sensing of alveolar pH. Biomedical optics express.

[16] Gu B, Yin M-J, Zhang AP, Qian J-W, He S (2009). Low-cost high-performance fiber-optic pH sensor based on thin-core fiber modal interferometer. Opt. Express.

[17] Shao L-Y, Yin M-J, Tam H-Y, Albert J (2013). Fiber optic pH sensor with self-assembled polymer multilayer nanocoatings. Sensors.

[18] Lee ST (2001). A sensitive fibre optic pH sensor using multiple sol-gel coatings. Journal of Optics A: Pure and Applied Optics.

[19] Mohamad F (2017). Controlled core-to-core photo-polymerisation - fabrication of an optical fibre-based pH sensor. Analyst.

[20] Ehrlich K (2017). pH sensing through a single optical fibre using SERS and CMOS SPAD line arrays. Opt. Express.

[21] MacCraith BD (1993). Fibre optic oxygen sensor based on fluorescence quenching of evanescent-wave excited ruthenium complexes in sol-gel derived porous coatings. Analyst.

[22] Chu C-S, Chuang C-Y (2015). Optical fiber sensor for dual sensing of dissolved oxygen and Cu2+ ions based on PdTFPP/CdSe embedded in sol–gel matrix. Sensors and Actuators B: Chemical.

[23] Vasylevska GS, Borisov SM, Krause C, Wolfbeis OS (2006). Indicator-Loaded Permeation-Selective Microbeads for Use in Fiber Optic Simultaneous Sensing of pH and Dissolved Oxygen. Chemistry of Materials.

[24] Kocincova AS, Borisov SM, Krause C, Wolfbeis OS (2007). Fiber-Optic Microsensors for Simultaneous Sensing of Oxygen and pH, and of Oxygen and Temperature. Analytical Chemistry.

[25] Shah VS (2016). Airway acidification initiates host defense abnormalities in cystic fibrosis mice. Science.

[26] Russell P (2003). Photonic crystal fibers. Science.

