# Supplementary material for: High fidelity fibre-based physiological sensing deep in tissue
Source: Sci Rep. 2019 May 22;9:7713. doi: 10.1038/s41598-019-44077-7 (PMC6531436; doi:10.1038/s41598-019-44077-7)
Supplement: Supplementary file 1 — Supplementary Dataset 1 [file 41598_2019_44077_MOESM1_ESM.pdf]

Supplementary Information for High fidelity fibre-based physiological sensing deep in tissue

Tushar R. Choudhary[1,2,*†], Michael G. Tanner[2,3,†], Alicia Megia-Fernandez[4], Kerrianne Harrington[5], Harry A. Wood[5], Adam Marshall[2], Patricia Zhu[4], Sunay V. Chankeshwara[4], Debaditya Choudhury[3], Graham Monro[2], Muhammed Ucuncu[4], Fei Yu[5], Rory R. Duncan[1], Robert R. Thomson[2,3], Kevin Dhaliwal[2], Mark Bradley[2,4]

†These authors contributed equally to this work

[1]Institute of Biological Chemistry, Biophysics and Bioengineering, School of Engineering & Physical Sciences, Heriot-Watt University, Edinburgh, UK

[2]EPSRC Proteus IRC Hub, Centre for Inflammation Research, Queen's Medical Research Institute, University of Edinburgh, Edinburgh, UK

[3]SUPA, Institute of Photonics and Quantum Sciences, School of Engineering & Physical Sciences, Heriot-Watt University, Edinburgh, UK

[4]*EaStChem,* School of Chemistry, University of Edinburgh, Edinburgh, UK

[5]Centre for Photonics and Photonic Materials, Department of Physics, University of Bath, Bath, UK

[*]Currently with The Roslin Institute and Royal (Dick) School of Veterinary Studies, University of Edinburgh, Edinburgh, UK

Reverse pH sensor

Figure S1 shows the effect of “saturated” and “standard” loading of FAM on the pH sensor response. Figure S1 A shows the response of “saturated” loaded pH sensors at two different pH values compared to “standard” loaded sensors shown in Figure S1 B. Corresponding longer wavelength emission is observed in a comparison of spectra with differing dye loading (Figure S1 C).

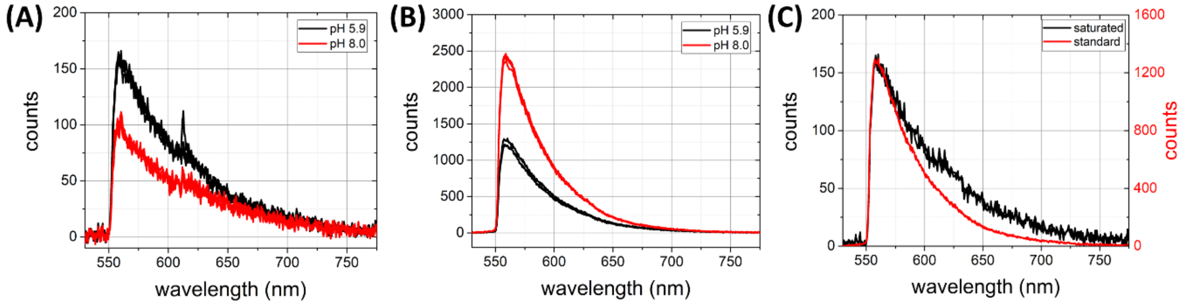

Figure S1. (A) FAM covalently attached to silica microsphere when “saturated” loaded decrease in fluorescence intensity with the increase in pH. (B) FAM covalently attached to silica microsphere when “standard” loaded increase in fluorescence intensity with increase in pH. (C) “saturated” (black line) and “standard” (red line) loading of FAM on silica microspheres have slightly different emission spectra. All the measurements were taken with 520 nm laser illumination (10 µW, 100 ms).

The “saturated” loading of FAM on silica microspheres was found to be advantageous as the resulting pH sensor was more robust to photobleaching (Figure S2 A) as compared to “standard” loading of FAM on silica microspheres (Figure S2 B). Repeated measurements at different powers were done in pH 7.0 buffer.

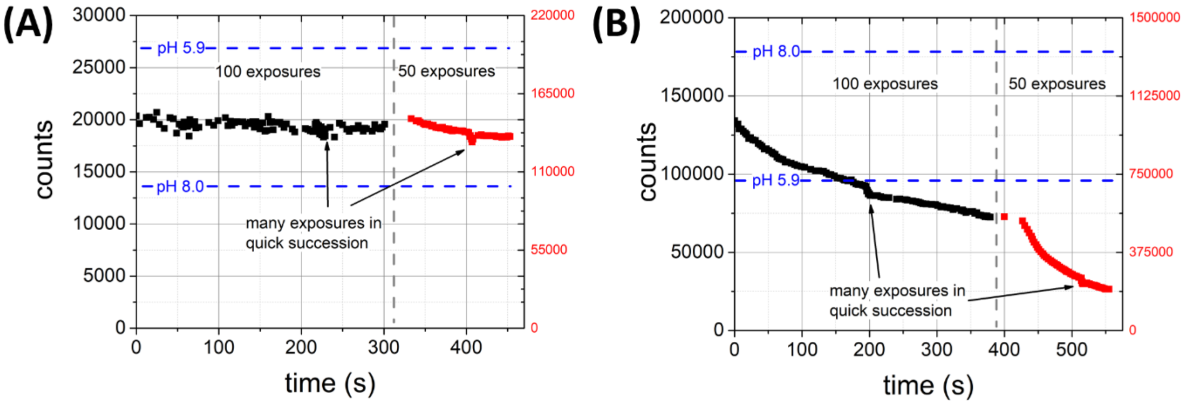

Figure S2. (A) “Saturated” loaded FAM pH sensor offering resilience to photobleaching at normal measurement powers (10 µW, black points), and some degradation at higher power (100 µW, red points). (B) “Standard” loaded FAM sensor showing much greater photobleaching under the same conditions. Representative response to pH is shown by the blue lines for relative scale, all measurements performed in pH 7.0 buffer. All the measurements were taken with 520 nm laser illumination.

Repeatability of optrode coupling

Repeatability of sensor loading was investigated through a survey of spectra from the fibre cores. Sensor signals from 4 different cores from multiple fibres for the pH sensor (Figure S3 A) and for the oxygen sensor (Figure S3 B) are shown. A repeatable sensor signal was obtained from different cores across multiple fibres for both the pH and oxygen sensor with some variation in amplitude likely due to varying sized microspheres with differing seating and therefore optical coupling in the cores. Distinct spectral characteristics allow identification of differing sensor locations.

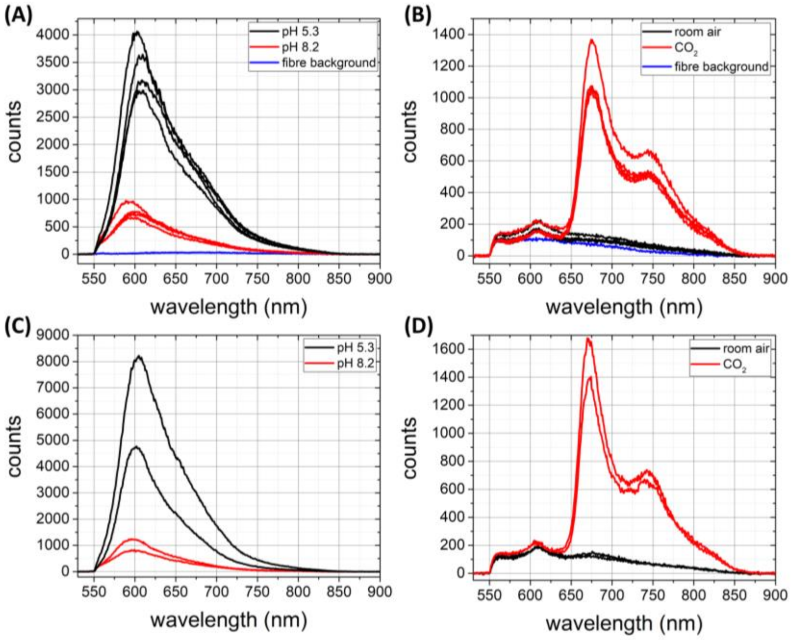

Figure S3. Spectra of pH and O2 sensors with varying conditions from multiple optrodes. All the measurements were taken with 520 nm laser illumination (10 µW, 100 ms). (A) shows signal from four different cores from two optrodes with pH sensors. Black lines represent spectra at pH 5.3 and red lines are the spectra obtained at pH 8.2. (B) shows signal from four different cores from two optrodes with oxygen sensors loaded. Red lines represent the spectra at no oxygen (depleted by CO_2) and black lines are the spectra obtained at room air (21% oxygen). Blue lines are the fibre background in the absence of any sensor. (C) and (D) show the corresponding responses from two cores of optrodes after EtO sterilisation.

Stability after sterilization

For clinical application, any sensing optrode must undergo and survive suitable sterilisation processes. The optrodes with pH and oxygen sensors were prepared and sterilised using an ethylene oxide (EtO) sterilisation process, which is commonly used for medical devices. The optrode performance was tested before and after sterilisation (Figure S3). A survey of cores was performed and spectra were recorded and compared pre and post sterilisation. The sensors survived the sterilisation regime used for clinical devices. Further tests such as toxicological and biocompatibility studies would be required in the future.
